# HemiBot: design and technical validation of an adaptive Robotic coaching system for Unilateral Spatial Neglect rehabilitation

**DOI:** 10.3389/frobt.2026.1950821

**Published:** 2026-10-21

**Authors:** Roberto Soldi, Bruna Maria Vittoria Guerra, Stefania Sozzi, Francesco Lunghi, Leo Russo, Micaela Schmid, Stefano Ramat

**Affiliations:** Bioengineering Laboratory, Department of Electrical, Computer and Biomedical Engineering, University of Pavia, Pavia, Italy

**Keywords:** adaptive difficulty, behavior tree, human-robot interaction, rehabilitation robotics, socially assistive robotics, TIAGo, Unilateral Spatial Neglect

## Abstract

**Introduction:**

Robot-mediated neuro-motor rehabilitation offers intensive, repeatable, and objectively monitored therapy, yet existing systems rarely integrate automated session management, real-time per-repetition scoring, and data-driven adaptive difficulty progression within a single non-contact architecture. This work presents HemiBot (Hemineglect roBot), a ROS 2 software stack implementing a fully automated coaching pipeline for semicircular arm-pointing exercises on the TIAGo mobile manipulator (PAL Robotics), targeting post-stroke Unilateral Spatial Neglect (USN), in which patients fail to orient toward or interact with stimuli in the contralesional hemispace.

**Methods:**

The robot’s end effector moves into the neglected hemispace as an exogenous attentional cue while the participant actively reaches toward it with the affected arm. The system extends a skeletal tracking framework with a Behavior Tree orchestrating exercise execution and per-repetition metric computation, a Coach node performing session-level adaptive difficulty progression, Italian empathetic text-to-speech feedback, and a scoreboard for therapist supervision. The scoring metric combines arm-alignment angle and hand–end-effector distance in a logical AND gate, yielding a normalized 0–100 score partitioned into three performance bands (Fail <25, Acceptable 25–75, Great ≥75). A participant is promoted when the mean session score exceeds 75 and demoted when the fail ratio reaches 0.50. Sixteen healthy participants (mean age 54.5 ± 6.8 years) were divided into two groups of eight: a high-performance group performing all repetitions correctly, and a low-performance group following a controlled error pattern designed to trigger demotion. Kinematic time series from the robot’s onboard RGB-D camera were compared against a stereophotogrammetric gold standard (BTS SMART-DX EVO).

**Results:**

Scoring concordance between TIAGo- and mocap-derived classifications reached 100% (Cohen’s κ = 1.000) across all 120 repetitions, with no cross-band errors between Fail and Great. The executed arc trajectory showed near-perfect agreement with the commanded geometry (Pearson r > 0.99). Adaptive behavior was correct for all 16 participants.

**Discussion:**

These findings verify that the onboard measurement chain reproduces gold-standard band assignments under the tested conditions and that the session-level decision pipeline operates end-to-end as specified. Validation of the scoring thresholds under naturally occurring, near-boundary performance remains necessary before clinical use.

## 1 Introduction

Unilateral Spatial Neglect (USN, or Hemineglect) is a frequent and disabling cognitive consequence of right-hemisphere stroke. Patients fail to orient toward, perceive, or act on stimuli in the contralesional (typically left) side of space, despite intact primary sensory and motor function (Bazan et al., 2022; Karner et al., 2019). The condition significantly prolongs hospitalisation, impedes rehabilitation, and is associated with poorer long-term functional outcomes (Winstein et al., 2016).

USN is a heterogeneous disorder that manifests differently depending on the spatial reference frame affected (Williams et al., 2021). In *egocentric* neglect, the patient fails to orient toward or interact with stimuli in the contralesional hemispace relative to their own body; in *allocentric* neglect, the patient ignores the contralesional side of individual objects, irrespective of their position in space (Marsh and Hillis, 2008). The two forms are clinically dissociable: egocentric neglect is by far the more prevalent form, affecting the large majority of USN patients as the sole or dominant subtype (Demeyere and Gillebert, 2019; Marsh and Hillis, 2008). Critically, egocentric neglect is the primary determinant of impairment in peripersonal activities–those requiring interaction with objects and stimuli within arm’s reach–such as reaching, object manipulation, and self-care (Williams et al., 2021).

At the neural level, USN arises from the dysfunction of distributed fronto-parietal networks rather than from focal cortical damage alone (Corbetta and Shulman, 2011; Bartolomeo, 2021). Damage to the right ventral fronto-parietal system disrupts both stimulus-driven reorienting and the broader network that controls voluntary spatial attention in body-centered coordinates, producing the characteristic egocentric spatial bias. Crucially, this network-based account opens a rehabilitative pathway: if intact nodes within the damaged network, or their homologs in the undamaged left hemisphere, can be recruited through appropriate sensorimotor activity, compensatory reorganisation becomes possible (Bartolomeo, 2021). Voluntary, goal-directed movement of the contralesional limb in contralesional space is one such activating strategy, engaging the same fronto-parietal circuits whose dysfunction underlies egocentric neglect.

Conventional therapies–visual scanning training, prism adaptation, and non-invasive brain stimulation–predominantly address the perceptual-attentional component of egocentric neglect and show variable transfer to functional activities of daily life (Bazan et al., 2022; Karner et al., 2019). Cueing-based interventions offer a complementary approach: graded, performance-driven adaptation of attentional cues has been shown to produce significant improvements in visuospatial neglect measures and activities of daily living (Turgut et al., 2018). The theoretical rationale for engaging the affected limb in USN rehabilitation can be framed within the *Where/Aiming* model (Barrett et al., 2022), which dissociates a perceptual-attentional deficit (*Where*, impaired orienting toward contralesional space) from a motor-intentional deficit (*Aiming*, impaired initiation and execution of movement directed into that space). The two components respond differentially to treatment (Barrett et al., 2022), yet most neglect treatment studies assess only one of them. Limb activation and spatiomotor cueing approaches addressed this gap by showing that voluntary movement of the contralesional limb within contralesional hemispace can facilitate spatial representation in the damaged hemisphere (Robertson and North, 1992; Kalra et al., 1997; Robertson et al., 2002). A recent systematic review further notes that the joint attentional-motor approach remains comparatively underexplored in robotic platforms (Somma et al., 2026). The semicircular pointing exercise operationalizes this principle through a robot-driven visual stimulus that specifically targets egocentric peripersonal neglect: the arc is centered on the participant’s left shoulder, placing the therapeutic stimulus in body-centered contralesional space within arm’s reach–the spatial domain whose disruption underlies the most functionally disabling egocentric neglect symptoms (Corbetta and Shulman, 2011; Verdon et al., 2010). The TIAGo end-effector moves into contralesional hemispace as an exogenous attentional cue, while the participant actively reaches toward it with the left arm, coupling the *Where* and *Aiming* components within a single non-contact task. Robot-assisted platforms have extended this principle to motor training. Exoskeleton-based systems such as the Armule (Chen et al., 2021) and manipulator-based platforms such as the REAplan (Doumas et al., 2025) deliver adaptive guidance through physical contact, achieving measurable motor gains. These systems, however, primarily target the motor execution component of neglect. Moreover, the paper-and-pencil assessments commonly used to evaluate their outcomes may fail to detect residual neglect that persists in everyday interaction with real three-dimensional objects (Bartolomeo, 2021). A recent systematic review confirms that robotic platforms remain underrepresented relative to virtual reality approaches and have rarely been designed to jointly capture attentional engagement and motor re-engagement within the same task (Somma et al., 2026).

Within the framework of the project described in (Guerra et al., 2025), we propose an additional, complementary paradigm: non-contact visual stimulation delivered through the TIAGo mobile manipulator. Previous work on TIAGo for motor-cognitive rehabilitation has relied on physical contact and natural language interaction (Tamantini et al., 2023); the present system adopts a fundamentally different approach, in which the robot’s end-effector serves as a moving attentional cue eliciting goal-directed pointing responses without any physical contact, targeting USN patients for whom exogenous visual cueing in the neglected hemispace is the primary therapeutic modality. The present work extends the robotic platform architecture previously described in Soldi et al. (2025), adding automated session management, per-repetition scoring, and adaptive difficulty progression to the existing exercise-delivery infrastructure.

A direct measure of the attentional (*Where*) component would require estimating where the participant is actually looking. In prior work (Soldi et al., 2025), we explored MediaPipe Face Landmarker (Google LLC, 2020a) to estimate head rotation relative to the end-effector as a proxy for gaze direction, analogous to the arm-alignment metric used here for the motor component. However, under the experimental conditions of that study this approach proved insufficiently robust, producing large estimation errors caused by self-occlusion of facial landmarks during head rotation. For this reason, the present system does not yet implement a *Where*-specific attentional measure, so that the results reported here focus on the motor-kinematic (*Aiming*) component described above. A direct measure of the *Where* component is nonetheless feasible in principle through wearable eye-tracking glasses, which record the wearer’s ocular movements and map the gaze point onto the scene: by verifying whether and when the participant’s gaze lands on the end-effector as it moves into the contralesional hemispace, such instrumentation would provide a continuous attentional variable analogous to the arm-alignment metric presented here for the motor component. This gaze-on-target signal is the attentional measurement modality we intend to integrate into HemiBot, and is discussed further in the Limitations and Future Directions sections.

A critical question remains open for any clinically deployable robotic coaching system: can it autonomously manage a full rehabilitation session, assess per-repetition performance with sufficient reliability, and adapt exercise difficulty to the participant’s evolving capability? These three capabilities are rarely integrated and systematically validated together in the existing literature, and their joint demonstration is a prerequisite for clinical deployment.

To enable autonomous, repeatable delivery of this paradigm, this paper presents *HemiBot* (Hemineglect roBot), a ROS 2 (Open Robotics, 2022) software stack that extends the TIAGo platform with a Behavior Tree for exercise execution and per-repetition scoring, a Coach node for adaptive difficulty progression, and Italian empathetic text-to-speech (TTS) feedback.

The contributions of this work are threefold:

1. A complete, modular ROS 2 software architecture for autonomous rehabilitation session management, described in sufficient detail to support reproducibility and future extension.
2. Validation of the per-repetition scoring metric against a stereophotogrammetric gold standard on 16 healthy participants across two sessions, quantifying the concordance between robot-derived and mocap-derived performance classifications.
3. Controlled demonstration of correct adaptive behavior under two distinct performance conditions (high-performance and low-performance), confirming that the promotion and demotion logic operates as designed.

The envisioned clinical deployment target is the Neurology Clinic *Fondazione IRCCS Mondino* in Pavia, with a trained therapist always present during sessions. The system is conceived as a tool to enhance therapeutic efficiency, not to replace clinical judgment.

The long-term objective underlying HemiBot is implementing a robotic system capable of autonomously managing a complete USN rehabilitation session, composed of multiple, heterogeneous exercise modalities, while continuously measuring patient performance and adapting task difficulty in real time. The present work addresses one constituent module of this broader architecture: a contactless, mobile visuo-motor stimulation exercise, together with its associated performance-scoring and adaptive-difficulty mechanisms. Accordingly, the study reported here should be considered as a technical validation of this specific module and of the underlying measurement/decision pipeline, through an experimental interaction with healthy volunteers. No clinical validation of the full multi-exercise session-management architecture, of its interaction with the target USN population and of its rehabilitative effectiveness was faced so far, but will be the object of future clinical trials.

## 2 Materials and methods

### 2.1 Exercise definition and difficulty levels

The rehabilitation exercise consists of a *semicircular arm-pointing task*: the TIAGo end-effector moves along a circular arc centered on the participant’s left shoulder, first sweeping rightward (from 0° to $+\theta_{\text{max}}$ ), pausing at the extreme, then sweeping leftward across the midline (to $-\theta_{\text{max}}$ ), pausing again, and returning to center. Angles are expressed in degrees relative to the anteroposterior axis, with forward defined as 0° and clockwise as positive.

Each repetition is decomposed into five consecutive segments:

1.
$0{}^{\circ}\to +\theta_{\text{max}}$ (rightward sweep)
2. Pause at $+\theta_{\text{max}}$ (dwell time $t_{\text{wait}}=2$ s)
3.
$+\theta_{\text{max}}\to -\theta_{\text{max}}$ (full crossing)
4. Pause at $-\theta_{\text{max}}$ (dwell time $t_{\text{wait}}=2$ s)

$-\theta_{\text{max}}\to 0{}^{\circ}$ (return to center)

Three difficulty levels are defined by varying $\theta_{\text{max}}$ (Table 1). All levels share the same arc radius ( r=0.85 m), end-effector angular velocity, and number of repetitions per session (3). Trajectory parameters are specified in a YAML configuration file loaded at runtime by the Coach node, making it straightforward to add or modify difficulty levels without code changes.

**TABLE 1:** Difficulty levels for the semicircular arm-pointing task. All levels share the same arc radius ( r=0.85 m), angular velocity, repetitions per session (3), and dwell time at extremes ( $t_{\text{wait}}=2.0$ s).

Level	$\theta_{\text{max}}$ (deg)	Arc extent (deg)
Easy	±10	20
Medium	±15	30
Hard	±20	40

### 2.2 Hardware platform

The experimental platform is the TIAGo mobile manipulator (PAL Robotics), a service robot featuring a mobile base, height-adjustable torso, 2-DOF head, and 7-DOF arm providing kinematic redundancy. Onboard sensors include an RGB-D camera and a 4-microphone array. The arm is equipped with a parallel gripper that can hold objects serving as visual stimulus. The full hardware description and technical specifications are provided in PAL Robotics (2026); the present description is limited to the software architecture built on top of this platform.

### 2.3 Software architecture overview

Two independent layers cooperate to manage a rehabilitation session:

- Session layer (Coach node): a ROS 2 node that loads the session plan from a YAML configuration file, sequences the exercises, dispatches triggers to the BT, collects session-aggregate results and displays them on a real-time graphical scoreboard visible to the experimenter, and decides the difficulty level for subsequent sessions.
- Execution layer (Behavior Tree): a ROS 2 BT node implemented in Python using the py_trees library (Stonier, 2025). It coordinates real-time exercise execution, kinematic monitoring, per-repetition metric computation, and emergency stop handling. This layer operates at the timescale of individual repetitions.

The graphical interface allows the therapist to monitor session progress in real time and, if needed, confirm or override individual repetitions before the BT advances. Empathetic feedback in Italian is delivered to the participant after each repetition *via* the/tts_engine/tts action server natively available on the TIAGo platform, with messages selected probabilistically from predefined pools corresponding to the three performance bands (Fail, Acceptable, Great). Representative examples of these messages are provided in the Supplementary Material.

### 2.4 TIAGo arm action server

The low-level arm control is handled by a custom C++ ROS 2 node, tiago_arm_action_server, implemented using the MoveIt 2 motion planning framework (PickNik Robotics, 2025). The node exposes two ROS 2 action interfaces:

- /tiago_arm/setup_pose (SetupPose action): brings the arm to a predefined joint-space configuration prior to each repetition, ensuring a consistent and ergonomically appropriate starting posture. Specifically, the setup pose corresponds to the neutral position of the semicircular trajectory ( 0° eccentricity), with the end-effector placed at the center of the arc in front of the participant. This guarantees that each repetition begins from a well-defined, reproducible arm configuration that is geometrically aligned with the subsequent motion.
- /tiago_arm/execute_motion (ExecuteMotion action): executes a Cartesian-space arc trajectory from the starting position using the semicircle motion type. The trajectory is parametrized by the arc center (the participant’s left shoulder), the radius r , and the start and end angles ( $\theta_{\text{start}}$ , $\theta_{\text{end}}$ ) relative to the anteroposterior axis. The arc is discretized into Cartesian waypoints spaced by a configurable angular step, with an optional dwell time $t_{\text{wait}}$ at each waypoint. The end-effector orientation is continuously adjusted so that the gripper always faces the arc center, preserving a consistent stimulus orientation at every eccentricity. Progress feedback (phase and completion ratio) is published at 5 Hz.

#### 2.4.1 Cartesian path planning and velocity profiling

Trajectory execution relies on MoveIt 2 Cartesian path computation (computeCartesianPath), which interpolates end-effector poses along the requested path while respecting joint limits and collision constraints. A custom trapezoidal velocity profile is then applied to the resulting joint trajectory: the profile is parameterized by the desired segment transition time and scaled by configurable maximum joint velocity and acceleration factors, ensuring smooth acceleration and deceleration at the beginning and end of each motion segment. Before execution, the trajectory is validated against joint velocity and acceleration limits; if any limit is violated the goal is aborted and an informative error is returned to the caller.

#### 2.4.2 Collision avoidance with dynamic patient model

The action server integrates a real-time patient collision model into the MoveIt 2 planning scene. The patient’s skeletal joint positions, published as a MarkerArray on/skeleton3d_markers by the monitoring node in the BT, are translated into spherical collision objects (radius $r_{\text{skel}}=0.10$ m) and applied to the PlanningSceneInterface at every incoming frame. Stale objects from the previous frame are removed before each update. Additionally, the OctoMap occupancy representation (Hornung et al., 2013), built directly from the raw depth stream independently of skeletal tracking, is cleared *via* the/clear_octomap service only at the start of each motion and is otherwise continuously repopulated during execution. Because it relies on depth measurements rather than skeleton keypoints, the OctoMap layer keeps marking the participant’s body volume as occupied even if the skeletal tracker temporarily loses or degrades its joint estimates, providing a coarser but tracking-independent fallback around the tighter, keypoint-driven spherical margin. Together, these two representations are intended to make the motion planner account for the participant’s current body configuration when computing each trajectory segment. At the trajectory level, the reaching targets themselves are designed *a priori* to keep the end-effector at a safe distance from the participant throughout the task, rather than relying on collision avoidance alone to prevent contact.

#### 2.4.3 Emergency stop and preemption

The action server monitors two Boolean flags, estop_active_ and cancel_requested_, checked at each iteration of the execution loop. An Emergency-Stop (E-Stop) message received on/protocol/session/stop sets both flags to true atomically and calls move_group_->stop() for immediate arm deceleration, causing the executing thread to terminate at the next loop iteration. New goals are rejected while estop_active_ is set; the flag is cleared only upon receipt of a resume message on/protocol/session/resume, after which clearPoseTargets() discards any residual MoveIt 2 state. The end-to-end responsiveness of the stop mechanism is bounded on the software side by the Behavior Tree tick rate: since EStopMonitor and the action-server execution loop are evaluated at 30 Hz, an E-Stop event is detected and move_group_->stop() is issued within one tick, i.e., approximately 33 ms in the worst case, before mechanical deceleration begins. We note, however, that this figure characterizes only the software detection-to-command latency; the additional hardware contribution, actuator command latency and the physical deceleration time and distance of the arm, was not experimentally measured in the present study. A dedicated characterization of the full stopping latency and stopping distance under representative motion conditions was outside the scope of the present study and is left to future safety validation.

### 2.5 Behavior Tree structure

The Behavior Tree (BT) has a two-level Parallel structure (Figure 1). In py_trees, a Parallel node ticks all children at every BT cycle, allowing multiple children to be in the running state simultaneously within the same thread. Crucially, regardless of the success policy, any failure returned by a child immediately propagates to the parent Parallel, which halts all sibling behaviors and returns failure (Stonier, 2025).

**FIGURE 1:**
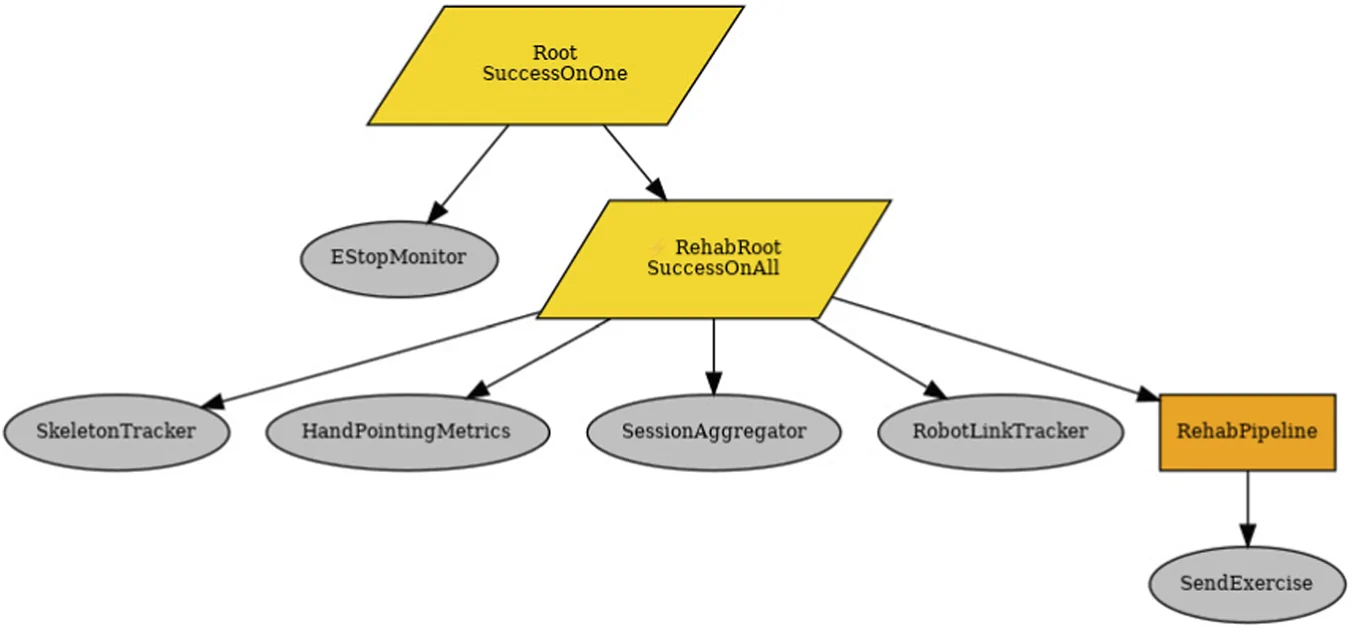
The root Parallel node (SuccessOnOne) runs the EStopMonitor safety leaf alongside RehabRoot, a second Parallel node (SuccessOnAll) that concurrently executes the perception and logging leaves–SkeletonTracker (body-joint tracking), HandPointingMetrics (real-time scoring), SessionAggregator (session-level aggregation), and RobotLinkTracker (robot kinematics logging) – together with the RehabPipeline subtree, which drives exercise execution through SendExercise.

The tree is designed to remain in running indefinitely under normal operation: EStopMonitor returns running as long as no stop signal is received, and SendExercise is programmed to return running at all times–including after the completion of a full exercise sequence, when it automatically resets and awaits the next trigger. This design ensures that neither the exercise pipeline nor the monitoring leaves ever terminate the tree through success; the only mechanism that can abort the session is EStopMonitor returning failure upon receiving a message on/protocol/session/stop. This failure propagates instantaneously through the root Parallel (SuccessOnOne), halting RehabRoot and all five of its children simultaneously–SkeletonTracker, HandPointingMetrics, SessionAggregator, RobotLinkTracker, and RehabPipeline–regardless of the current phase of exercise execution. The SuccessOnOne and SuccessOnAll policies on the two Parallel nodes therefore define the nominal success semantics of the tree, but under normal operation neither is triggered: session termination is exclusively event-driven through EStopMonitor.

1. SendExercise: waits for a JSON trigger on the topic/protocol/exercise/execute, commands the TIAGo arm action server to execute the prescribed trajectory sequence for each repetition, and publishes repetition results to/protocol/exercise/repetition_result. After each repetition it pauses and awaits confirmation (/protocol/exercise/agent_feedback) before advancing. This node manages the entire repetition lifecycle: setup pose, motion execution, result publication, and repeat/abort logic.
2. RobotLinkTracker: records the robot’s kinematic state throughout the session to enable post-hoc validation of the motion control pipeline. Subscribing to/joint_states and querying the TF tree for the end-effector pose in the *base_footprint* frame, it timestamps and logs, at a fixed rate, both the commanded and the measured joint trajectories together with the corresponding Cartesian pose of the gripper. Data are optionally serialized to CSV files, allowing offline analysis.
3. SkeletonTracker: performs three-dimensional, markerless tracking of the participant’s body joints, publishing the results as TF frames consumed by HandPointingMetrics. RGB and depth streams are processed with MediaPipe Pose Google LLC (2020b) to extract landmarks, which are back-projected into 3D *via* the pinhole camera model OpenCV (2026) and transformed into the *base_footprint* frame using the URDF (Unified Robot Description Format) kinematic chain. Only four joints (shoulders and wrists) are tracked to limit data volume and occlusion issues, with a 3D Kalman filter smoothing the trajectories. The 3D Kalman filter smooths the per-frame landmark estimates produced by the markerless pipeline, which are intrinsically noisy; it is not used to bridge missing observations by prediction. MediaPipe’s per-landmark visibility score is recorded alongside each estimate but is not currently used as an explicit confidence threshold, and no explicit occlusion (or self-occlusion) detection is performed. The only criterion for discarding a sample is the depth reading: a landmark is treated as missing for a given frame whenever the corresponding pixel yields an invalid or unavailable depth value, in which case the sample is omitted rather than replaced by a filtered or extrapolated estimate. Occlusions that still yield a valid depth reading are therefore retained and may introduce artifacts in the tracked trajectory, an effect not addressed in the present work.
4. HandPointingMetrics: monitors the participant’s kinematic response in real time by reading TF frames published by SkeletonTracker. It implements a three-state machine (idle → recording → processing) synchronized with the exercise status on the BT blackboard. During recording, it accumulates metric samples at each BT tick (30Hz). At processing, it computes the per-repetition score (Section 2.6) and writes the results to the blackboard. Optionally, it saves full kinematic timeseries as CSV files and publishes them on/protocol/exercise/kinematics_timeseries.
5. SessionAggregator: collects per-repetition metrics from the blackboard whenever a repetition is validated, accumulates them in an internal history, and computes session-aggregate statistics (mean score, mean arm misalignment, mean hand distance, mean duration). At session end, it publishes a structured JSON report on/protocol/exercise_result with TRANSIENT_LOCAL QoS, ensuring the Coach node receives the report even if it subscribes after publication. The JSON report is self-contained and schema-consistent across sessions, making it directly suitable for ingestion into a longitudinal participant database without further post-processing.
6. EStopMonitor: polls the emergency stop status at each tick. If an E-Stop event is detected, it sets a flag on the blackboard and returns failure, causing the Parallel node to abort all sibling behaviors and terminate the session. This mirrors the preemption logic implemented in the action server (Section 2.4), ensuring that an E-Stop event is handled consistently at both the motion control level and the session orchestration level.

The Parallel structure guarantees that kinematic monitoring and aggregation remain active throughout the entire session, regardless of the state of exercise execution, without requiring explicit synchronisation between nodes.

### 2.6 Per-repetition scoring metric

Task performance for each repetition is quantified by a normalized score S∈[0,100] computed from two kinematic indicators, both validated against a stereophotogrammetric reference system in the present study (Section 3.1):

- Arm alignment angle α (degrees): the angle between the segment connecting the participant’s wrist to their shoulder and the segment connecting the robot end-effector to the participant’s shoulder. A smaller α indicates more accurate pointing.
- Hand–end-effector distance d (meters): the Euclidean distance between the participant’s wrist and the robot end effector. While α captures whether the participant is pointing in the correct direction, d specifically captures whether the participant also extends the arm toward the target, so that a small d indicates genuine reaching rather than directional pointing alone.

The two indicators are evaluated against fixed threshold values ( $\alpha_{\text{th}}=10{}^{\circ}$ , $d_{\text{th}}=0.50$ m) using a logical and gate: a sample is classified as *on-target* only if both conditions are satisfied simultaneously. The and gate prevents false positives arising from cases in which the arm points in the correct direction but does not reach the target, or *vice versa*. The score is computed as the proportion of on-target samples within the interval of interest for each repetition as defined in Equation 1:

$$S=100\times \frac{\sum_{k=1}^{N}1\;\left[\alpha_{k}\leq \alpha_{\text{th}}\;\wedge \;d_{k}\leq d_{\text{th}}\right]}{N}$$ (1)

where N is the total number of samples in the interval and 1[⋅] is the indicator function. The score is evaluated over the entire duration of the repetition, from the initial setup pose to the return to the center, with samples acquired at the camera frame rate ( ≈30 Hz) by the HandPointingMetrics node.

Three performance bands partition the score range:

-
S<25 : Fail–the repetition is rejected and must be repeated.
-
25≤S<75 : Acceptable–the repetition is validated; the participant proceeds.
-
S≥75 : Great–the repetition is validated as high-quality performance.

### 2.7 Adaptive difficulty progression

After each session, the Coach node reads the aggregate report published by SessionAggregator and applies the decision rules in Table 2 to determine the difficulty level for the subsequent session. Demotion takes precedence over promotion: if both conditions are simultaneously satisfied (e.g., due to a high score on the few successful attempts but a high overall fail ratio), the demotion rule is applied.

**TABLE 2:** Adaptive difficulty progression rules. $\bar{S}$ : mean score over all repetitions attempted in the session, including those classified as Fail. $r_{\text{fail}}$ : ratio of failed attempts (including retries) to total attempts. Demotion takes precedence over promotion.

Condition	Action	Rationale
$r_{\text{fail}}\geq 0.50$	Demote to previous level	Session predominantly failed
$\bar{S}\geq 75$	Promote to next level	Consistent high performance
$25\leq \bar{S}<75$	Maintain current level	Acceptable, consolidate

The progression rules, thresholds, and initial difficulty level are all configurable in the YAML session plan. At the end of each repetition, the system delivers immediate qualitative feedback to the participant *via* a TTS message in Italian, selected probabilistically from predefined pools corresponding to the three performance bands (Fail, Acceptable, Great). After determining the next difficulty level, the Coach node additionally synthesizes a session-level TTS message selected from three pools corresponding to the demotion, maintain, and promotion outcomes, informing the participant of the session result and the plan for the next session. Figure 2 presents the therapist’s view of the scoreboard, showing the results generated by the system, including scores, categories and output utterances.

**FIGURE 2:**
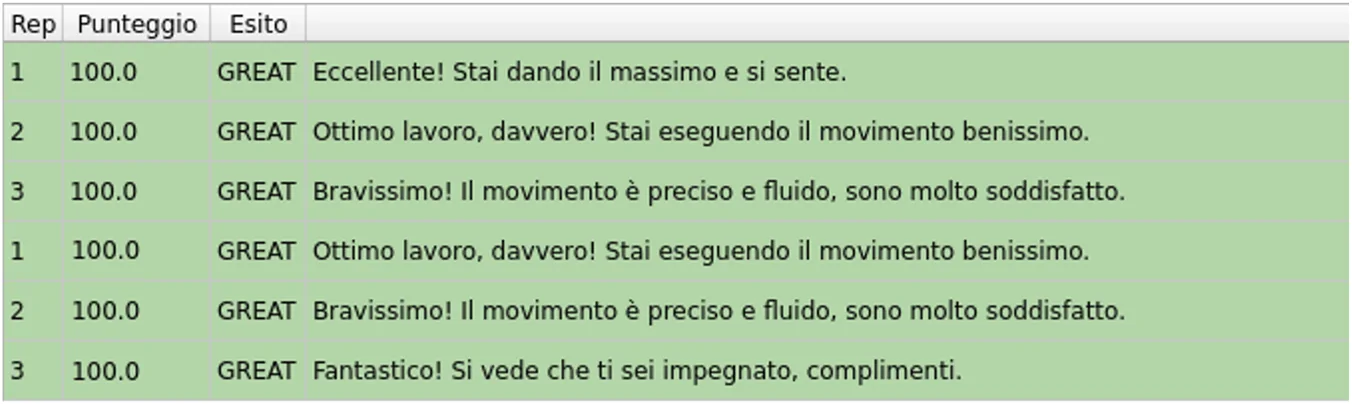
Per-repetition feedback generated by the Coach node for a representative subject across two consecutive sessions (three repetitions each). For every repetition, the table reports the repetition index (*Rep*), the numeric score (*Punteggio*), the qualitative outcome (*Esito*), and the corresponding natural-language feedback message given to the participant. In this example, all six repetitions across both sessions achieved the maximum score (100.0, GREAT), with the system varying the feedback phrasing between repetitions to avoid repetitive, mechanical-sounding encouragement.

The performance band boundaries ( S=25 , S=75 ) were derived from clinical practice in consultation with the rehabilitation specialists at Fondazione IRCCS Mondino, and are consistent with adaptive difficulty frameworks established in robot-assisted rehabilitation: a target success rate of approximately 70% has been identified as optimal for balancing motivation and challenge, with session-level demotion triggered when performance falls substantially below this range (Metzger et al., 2014), analogous to the concept of proximal development zone in which a task is neither trivially easy nor beyond reach (Vygotsky, 1978). This is also in line with the Challenge Point framework (Guadagnoli and Lee, 2004), according to which learning is optimized when functional task difficulty is matched to the performer’s skill level: a task that is too easy conveys little corrective information and one that is too difficult overwhelms the learner’s capacity to interpret feedback, so an intermediate, sustained band of performance (here, the Acceptable range 25≤S<75 ) is expected to support consolidation rather than triggering an immediate change in difficulty. The specific value of the upper threshold is further supported by a recent clinical validation of a dynamic-difficulty-adjustment mechanism for combined motor-cognitive robotic rehabilitation after stroke, which adopted a reference success rate of 75% as the target for balancing motivation and learning, and used deviations from this target to drive difficulty adaptation in a manner directly analogous to the promotion/demotion logic used here (Doumas et al., 2025). The upper boundary (S≥75) therefore indicates consistent mastery of the current level and warrants progression, the lower boundary (S<25) indicates a task that exceeds the participant’s current capability, and the intermediate band (25≤S<75) corresponds to the consolidation range in which further practice at the same level is expected to be beneficial.

A geometrical interpretation can be given to the scoring thresholds: at the nominal arm length of r=0.85 m, an angular error of $\alpha_{\text{th}}=10{}^{\circ}$ corresponds to a linear displacement between the hand and the target of

$$d=2r\sin\;\left(\frac{\alpha_{\text{th}}}{2}\right)=2\times 0.85\times \sin\left(5{}^{\circ}\right)\approx 0.148\text{ m}$$ (2)

a tolerance of approximately 15 cm at the nominal arc length (Equation 2), reflecting the residual motor impairment expected in the target patient population while still requiring accurate directional pointing.

### 2.8 Experimental protocol

#### 2.8.1 Participants

Sixteen healthy adult volunteers were enrolled in the study (9 female, 7 male; mean age 54.5±6.8 years). Stroke predominantly affects adults over 50 years of age, with prevalence and incidence rising sharply with age (He et al., 2024); the recruited age range was therefore deliberately chosen to approximate the demographic profile of the intended clinical target population, namely, post-stroke patients in the subacute or chronic phase. This choice increases the ecological validity of the normative thresholds derived from the control sample with respect to future clinical use. Participants were assigned to one of two groups of eight (Group A: *high-performance*, Group B: *low-performance*). All participants provided written informed consent in accordance with the Declaration of Helsinki.

#### 2.8.2 Apparatus and setup

Participants were seated at a fixed distance of 1.55 m from the TIAGo reference frame (base_footprint). The arc radius was set to r=0.85 m for all participants. Note that the seating distance of 1.55 m refers to the position of the participant’s torso relative to the robot’s base_footprint (the center of the robot base), whereas the arc radius of 0.85 m is the distance between the robot’s end-effector and the participant’s left shoulder at the nominal setup pose. Kinematic ground truth was provided by a BTS SMART-DX EVO stereophotogrammetric motion capture system (BTS Bioengineering, 2026). Two markers were placed on the gripper to recover the gripper_grasping_frame. Four markers were placed on the participant (bilateral wrists and shoulders). The robot’s onboard RGB-D camera simultaneously acquired data for markerless skeletal tracking *via* MediaPipe Pose Google LLC (2020b). The rigid-body rototranslation between the mocap coordinate frame and the robot’s base_footprint frame was computed from three markers placed on anatomically identifiable points of the TIAGo chassis: base_antenna_left_link, base_antenna_right_link, and torso_lift_link. These landmarks were selected because they correspond to accessible flat surfaces of the robot body, allowing precise and repeatable marker placement across sessions. The transformation was estimated as a standard rigid rototranslation (rotation matrix R and translation vector t ) minimising the residual between the known positions of the three points in the robot frame and their mocap measurements, and was applied to all BTS-derived coordinates prior to analysis.

For each trial, the residual error of the rigid rototranslation was quantified as the discrepancy between the known chassis-marker positions in the robot frame and their transformed mocap counterparts. Across all trials, the mean residual error was 5.8±1.3 mm (mean ± SD of the per-trial mean residual), confirming sub-centimetre accuracy of the BTS-to-TIAGo frame registration and establishing the accuracy floor for all subsequent kinematic comparisons (see also Section 3.1). The accuracy of the shoulder-relative arc centering assumption (Section 2.4) was independently verified against the BTS reference. The nominal setup pose places the end-effector 0.85 m in front of the participant’s left shoulder (0 m laterally, corresponding to 0° eccentricity). Across all participants, the BTS-measured shoulder position relative to the initial end-effector pose was of 0.869±0.073 m (mean ± SD). This confirms that the fixed seating protocol (Section 2.8.2) reliably approximates the nominal arc center across participants.

### 2.9 Session protocol

Each participant completed two experimental sessions in sequence. Throughout both sessions, participants were guided by the experimenter on how to perform the task: to actively reach toward the robot’s end-effector with the left arm, following its movement along the arc. The difficulty level of the second session was automatically assigned by the system based on the aggregate performance outcome of the first.

#### 2.9.1 High-performance group (n=8)

Session one consisted of three medium-difficulty repetitions (±15°) , all performed correctly and validated by the robot with scores ≥75 . The resulting mean session score $\bar{S}\geq 75$ and the fail ratio equal to 0 triggered automatic promotion to the hard level. Session 2 consisted of three hard-difficulty repetitions (±20°) , performed correctly.

#### 2.9.2 Low-performance group (n=8)

Session one consisted of six medium-difficulty repetitions (±15°) following a controlled error pattern: one correct repetition (score ≥75 ), two failed repetitions (score =0 ), one partial repetition (score ≈50 ), one failed repetition (score =0 ), and one final correct repetition (score ≥75 ). Failed repetitions (score =0 ) were produced by participants keeping their arms resting on their laps throughout the trial, resulting in no samples satisfying the AND-gate condition. The partial repetition (score ≈50 ) was produced by following the end-effector only along the rightward half of the arc (from center to +15° and back to center), then keeping the arm at rest during the leftward excursion into the contralesional hemispace ( 0° to −15° ), emulating the attentional behavior expected in USN patients who fail to engage with stimuli presented on the neglected side. Three out of six attempts were failed (fail ratio ≥0.50 ), triggering automatic demotion to the Easy level. Session 2 consisted of three Easy-difficulty repetitions (±10°) , all performed correctly. The error pattern in session one was deliberately piloted by the experimenter and was not intended to reflect the subjects’ actual motor capabilities. The purpose was to systematically verify the correct operation of the demotion logic under a controlled condition: this validation therefore demonstrates that the adaptive-difficulty algorithm correctly detects and responds to a known, scripted sequence of performance outcomes, rather than emulating the natural, continuous, and often inconsistent performance variability expected from real USN patients across repetitions and sessions. Assessing how the algorithm behaves under such organic variability, particularly for performance values close to the promotion/demotion thresholds, is left to future clinical validation.

In total, this protocol yielded 120 recorded repetitions: 48 from Group A (8 participants × three repetitions per session) and 72 from Group B (8 participants × six repetitions in Session 1, plus three in Session 2).

### 2.10 Data collection and analysis

For each repetition, full kinematic timeseries of arm alignment angle α(t) , hand–end-effector distance d(t) , and 3D end-effector position were computed from both the robot’s onboard system and the BTS mocap reference. Per-repetition aggregate metrics (score S , mean $\bar{\alpha }$ , mean $\bar{d}$ , duration) were logged by the SessionAggregator node.

Four analyses were performed:

1. Kinematic metric accuracy: RMSE was computed between TIAGo-derived and mocap-derived timeseries of α and d . The RMSE analysis targets the agreement between the two measurement instruments (TIAGo’s onboard estimate *versus* the mocap gold standard). Since the measurement error of a sensing pipeline is not expected to depend on which participant is being tracked, individual repetitions across participants were pooled and treated as the unit of analysis without stratifying by subject. Kruskal-Wallis comparisons were run on the 88 trials in which the subject was asked to execute the task correctly. The analysis of all 120 trials, instead, evaluated the trajectory of the end-effector, i.e., its radius of curvature, angular velocity, and instantaneous eccentricity θ(t) , to characterize the fidelity of the executed Cartesian arc trajectory beyond the scoring-relevant metrics. The trajectory radius of curvature was estimated for each repetition by projecting the end-effector 3D positions onto the horizontal (XY) plane and fitting a circle *via* an algebraic least-squares circle fit, which solves the linear least-squares system $2x\;c_{x}+2y\;c_{y}+c=x^{2}+y^{2}$ for the circle center $\left(c_{x},c_{y}\right)$ and the auxiliary term c , from which the radius is recovered as $r=\sqrt{c_{x}^{2}+c_{y}^{2}+c}$ .
2. Physiological pointing threshold: to assess whether the *a priori* scoring thresholds ( $\alpha_{\text{th}}=10{}^{\circ}$ , $d_{\text{th}}=0.50$ m, Section 2.6) are conservative with respect to genuine, correctly executed pointing responses, BTS-derived arm-alignment angle and hand–end-effector distance were extracted during all repetitions of the high-performance group (correct by design) and all session 2 repetitions (BTS score =100 ). For each metric, the 95th percentile of the pooled BTS distribution was taken as a data-driven estimate of the physiological upper bound on the alignment error expected during a genuinely correct pointing response.
3. Scoring concordance: for each of all 120 repetitions, spanning the Fail, Acceptable, and Great conditions by design, the score S computed from TIAGo-derived metrics was classified into the three bands (Fail/Acceptable/Great). The same classification was derived from mocap-based metrics using identical thresholds and formula. Unlike the kinematic accuracy analysis above, this analysis uses the complete set of 120 repetitions, since its purpose is to verify that the onboard scoring pipeline assigns each repetition to the correct performance band across the full range of intended behaviors, not only under correct execution. A 3×3 confusion matrix was constructed, and overall accuracy, Cohen’s kappa (Cohen, 1988), and per-class recall were reported.
4. Adaptive behavior validation: the difficulty level proposed by the system for session 2 was compared against the expected outcome for each participant, based on the session one aggregate statistics ( $\bar{S}$ and $r_{\text{fail}}$ ) and the decision rules in Table 2. Correct operation was defined as an exact match between the system-assigned level and the expected level for all 16 participants.

## 3 Results

### 3.1 Kinematic metric accuracy

Figure 3 summarizes the kinematic accuracy of the TIAGo onboard markerless pipeline relative to the BTS SMART-DX EVO stereophotogrammetric reference, computed on the 88 out of 120 total repetitions in which participants achieved a BTS-derived score of 100% (i.e., fully correct executions according to the gold-standard reference). The inter-system registration residual was 5.8±1.33 mm (mean ± SD, max 7.05 mm), establishing the accuracy floor of the BTS-to-TIAGo frame alignment.

**FIGURE 3:**
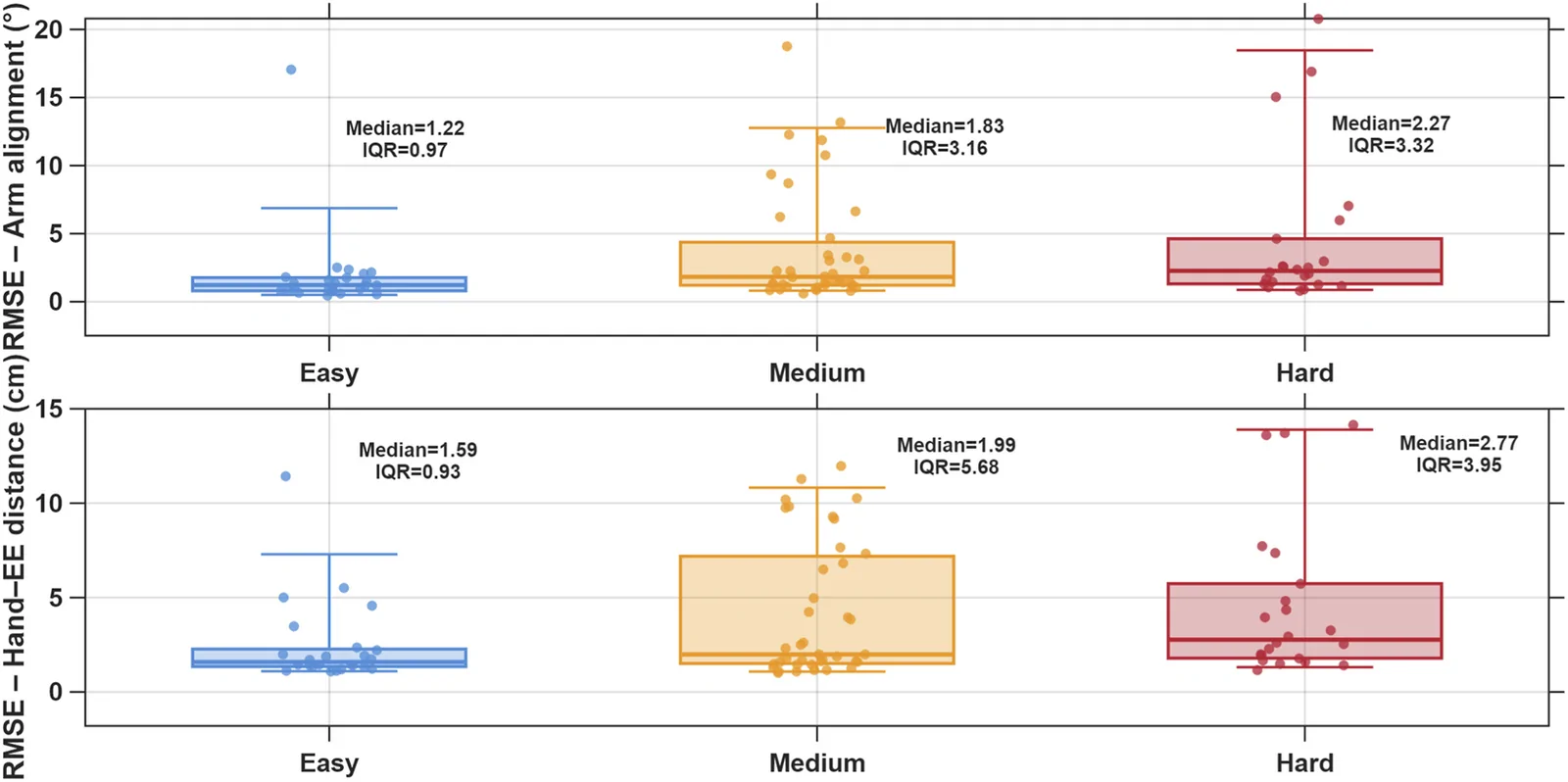
RMSE between TIAGo onboard measurements and BTS SMART-DX EVO reference measurements, for arm-alignment angle (top) and hand–end-effector distance (bottom), computed on the 88 repetitions in which the BTS-derived score was 100%, stratified by difficulty level (Easy, Medium, Hard). Individual trial values are overlaid as dots; boxes span the interquartile range; the central line indicates the median; whiskers extend to the 5th and 95th percentiles.

RMSE showed a statistically significant increase with arc eccentricity for both metrics. For arm alignment, values rose from a median of 1.22° (IQR 0.97° , n=24 ) at Easy to 1.83° (IQR 3.16° , n=40 ) at Medium and 2.27° (IQR 3.32° , n=24 ) at Hard (Kruskal-Wallis $\chi^{2}=11.01$ , p=0.004 ; Easy vs. Medium p=0.019 , Easy vs. Hard p=0.006 , Medium vs. Hard p=1.000 , Bonferroni-corrected). For hand–end-effector distance, RMSE increased from 1.59 cm (IQR 0.93cm, n=24 ) at Easy to 1.99 cm (IQR 5.68cm, n=40 ) at Medium and 2.77 cm (IQR 3.95cm, n=24 ) at Hard (Kruskal-Wallis $\chi^{2}=7.27$ , p=0.026 ; Easy vs. Medium p=0.162 , Easy vs. Hard p=0.026 , Medium vs. Hard p=0.899 ). Although median RMSE values remain modest across all levels, the elevated IQR at Medium and Hard difficulty reveals a subset of trials with substantially higher errors. These outliers are attributable to transient occlusions of the wrist landmark during lateral arm extension–a known failure mode of monocular markerless pose estimation (Dill et al., 2023) – which introduce brief but sharp deviations in the TIAGo-derived signal. Such events, while infrequent, can transiently affect both the kinematic metrics and the per-repetition score computed from them; their impact on classification is discussed in Section 3.3. The post-hoc comparisons show a consistent pattern for both metrics: the significant difference lies between Easy and the lateral levels (Medium and Hard), while the step from Medium to Hard is not significant, suggesting that the dominant error contribution arises from the transition out of the frontal workspace rather than accumulating linearly with eccentricity. The executed trajectory closely matched the commanded semicircular arc: the mean radius of curvature estimated from gold-standard measurements was 0.815±0.012 m (mean ± SD), corresponding to a systematic underestimate of 4.1% with respect to the commanded radius r=0.85 m, which is attributable to chord interpolation between discrete Cartesian waypoints. End-effector angular velocity showed near-perfect agreement between TIAGo and BTS across all repetitions (mean Pearson r=0.9975±0.0008 ; mean RMSE =0.20±0.05° /s), as did the instantaneous trajectory eccentricity θ(t) (mean r=0.9999±0.00003 ; mean RMSE =1.95±0.34° ). These results confirm that the TIAGo onboard kinematic pipeline faithfully reproduces both the geometry and the dynamics of the executed arc trajectory, independently of the scoring-relevant alignment and distance metrics reported above.

### 3.2 Physiological pointing threshold

To verify that the *a priori* scoring thresholds are conservative with respect to genuinely correct pointing responses, the arm-alignment angle and hand–end-effector distance were analyzed during all repetitions independently verified to be fully correct (Section 2.10). The 95th percentile of the pooled BTS gold-standard distribution was 4.6° for the arm alignment angle and 36.1 cm for the hand–end-effector distance, capturing the baseline physiologic variability of unimpaired pointing responses. Both values fall comfortably below the system’s *a priori* thresholds ( $\alpha_{\text{th}}=10{}^{\circ}$ , $d_{\text{th}}=0.50$ m), confirming that the scoring thresholds are appropriately conservative and unlikely to penalize genuine, on-target pointing performance.

The physiological distance value is also anatomically plausible rather than arbitrary. With the participant’s shoulder positioned at approximately 0.85 m from the end effector at the nominal reach configuration, the residual 36.1 cm reflects the sum of several geometric offsets between the two tracked reference points, not a coincidence-point error tolerance: the wrist marker used to compute d is located approximately 15 cm proximal to the fingertip along the hand, the reference point used for the end effector is located approximately 10 cm proximal to the tip of TIAGo’s gripper, and a further small clearance remains between fingertip and gripper even at genuine, correctly executed contact. This geometric decomposition indicates that 36.1 cm is a physically plausible upper bound for the wrist–end-effector residual distance during genuine interaction, further supporting the adequacy of the 0.50 m operational threshold, which retains a comfortable margin above this physiological estimate.

### 3.3 Scoring concordance

Figure 4a shows the per-repetition score distributions for both the TIAGo and BTS systems across both groups and sessions. In the high-performance group (Group A), scores from both systems were tightly concentrated near 100 in both sessions, confirming that participants performed the task correctly and that TIAGo and BTS metrics overlap closely. In the low-performance group (Group B), session one scores exhibited a trimodal distribution reflecting the controlled error pattern: a cluster at S≈0 (arms resting on laps, three repetitions per participant), a cluster at S≈50 (partial attempts matching the contralesional neglect profile; one repetition per participant), and a cluster near S=100 (correct attempts, two repetitions per participant). TIAGo- and BTS-based classifications demonstrated full agreement across all three performance clusters. Following automated system demotion, session 2 scores for the low-performance group converged to 100 for both systems, indistinguishable from those of the high-performance group at their respective difficulty level. The session-level trajectories underlying these transitions are shown in Figure 4b.

**FIGURE 4:**
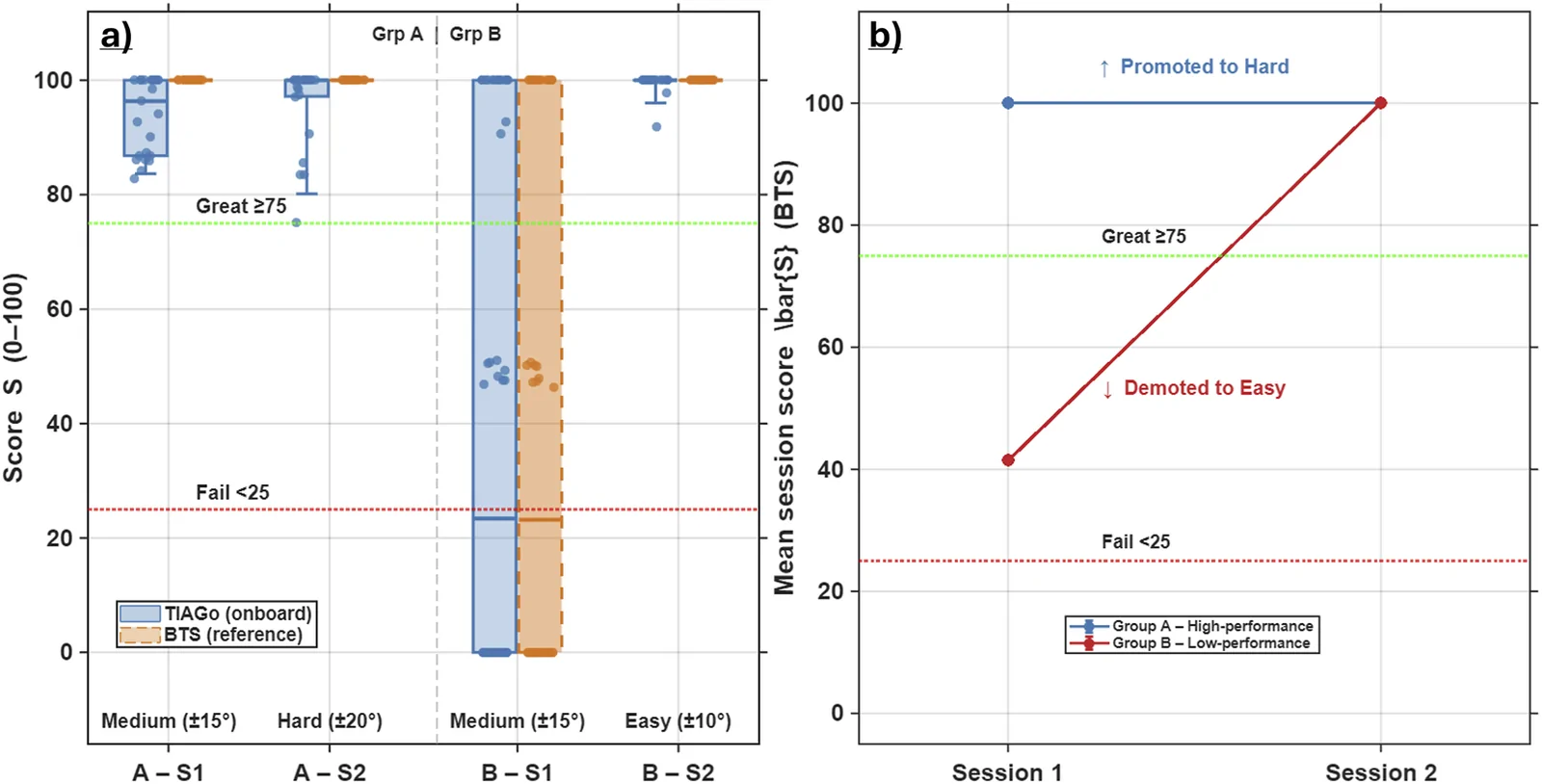
**(a)** Per-repetition scores S (0–100 scale) derived from TIAGo onboard measurements (blue, solid boxes) and BTS mocap reference (orange, dashed boxes), stratified by group and session: Group A (high-performance) in sessions one and 2, and Group B (low-performance) in sessions one and 2. The difficulty level of each condition is indicated below the x-axis. Horizontal dotted lines mark the performance band boundaries ( S=25 , Fail; S=75 , Great). Individual trial values are overlaid as dots. **(b)** Session-level mean scores ( $\bar{S}$ , BTS reference) for Group A (blue) and Group B (red) across the two sessions. Arrows indicate the difficulty-level transition automatically applied by the Coach node between sessions: Group A was promoted to the Hard level, Group B was demoted to the Easy level.

Beyond the aggregate RMSE values, Figure 5 illustrates this agreement at the level of the raw continuous signal for a representative repetition. Both the TIAGo-derived and mocap-derived traces of α(t) and d(t) closely track one another throughout the movement, with deviations remaining small relative to the margin separating them from the *a priori* scoring thresholds. This continuous-level view complements the pooled RMSE statistics and the discrete band-classification analysis (Section 3.3) by directly showing that the two instruments agree not only in their final classification outcome, but throughout the time course of the movement.

**FIGURE 5:**
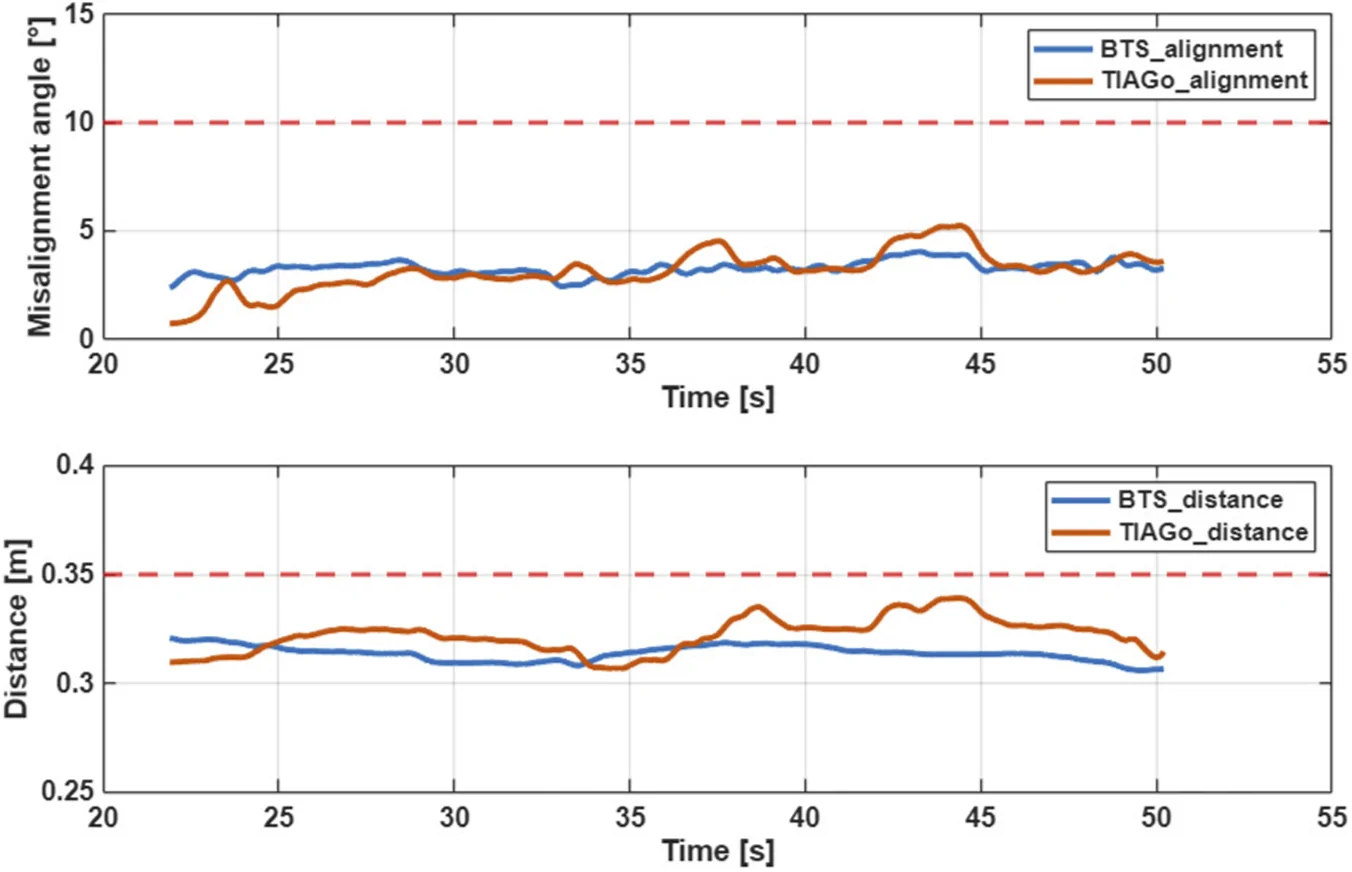
Continuous agreement between TIAGo-derived and mocap-derived kinematic signals for a representative repetition. Top: misalignment angle α(t) , with the dashed line marking the operational threshold $\alpha_{\text{th}}=10{}^{\circ}$ . Bottom: hand–end-effector distance d(t) ; the vertical axis is zoomed to a range that keeps the signals visible; the dashed line here marks the data-driven physiological reference bound ( ≈0.35 m, close to the 36.1 cm estimate reported in Section 3.2) rather than the operational scoring threshold ( $d_{\text{th}}=0.50$ m), which lies outside the plotted range. The two measurement systems track each other closely throughout the repetition, remaining well below both the physiological reference and the (off-scale) operational threshold at all times, illustrating the continuous-level agreement underlying the perfect discrete band-classification accuracy reported in Section 3.1.

Table 3 reports the 3×3 confusion matrix for per-repetition band classification (Fail/Acceptable/Great) comparing TIAGo-derived and mocap-derived scores across all 120 repetitions. The overall classification accuracy was 100% (Cohen’s κ=1.000 ). Although transient occlusion artifacts occasionally cause the TIAGo-derived score to deviate from the mocap-derived value within a repetition, these deviations remain confined within the same performance band in all 120 cases. A plausible source of increased occlusion in correctly executed repetitions is the proximity of the wrist to the end effector during active reaching: when the hand approaches the target, the end effector may partially occlude the wrist landmark in the RGB-D camera view. This effect is expected to be more pronounced in Group A, whose participants actively followed the end effector throughout the entire arc, maximizing the time spent with the wrist near the target. Consistently, the score deviations visible in Figure 4a are concentrated in Group A at Medium difficulty–the condition combining correct full-range reaching with a laterally extended arc for the first time. In contrast, the central cluster of Group B session 1 – comprising the partial repetitions (S≈50) , in which participants followed the end effector only along the ipsilesional half of the arc and kept the arm at rest during the contralesional excursion–shows tighter agreement between TIAGo and BTS scores, consistent with reduced wrist-to-end-effector proximity and therefore fewer occlusion events. As visible in Figure 4a, only one repetition approached the classification boundary, with a score near the Great threshold (S=75) ; all other cases were classified well within their respective bands. Critically, no cross-band errors between Fail and Great occurred in any of the 120 repetitions–the only class of error capable of triggering an erroneous adaptive decision. Given the wide separation of the tested conditions from the band boundaries, this result supports the robustness of the metric to the transient measurement artifacts identified in Section 3.1, but does not by itself establish its discriminative power under naturally graded performance. Notably, despite the experimental design deliberately targeting performance clusters well separated from the decision boundaries, the score of this single repetition nonetheless drifted close to the Great/Acceptable boundary, consistent with the wrist-occlusion effect already discussed above (Figure 4a): the proximity of the wrist to the end effector during active reaching intermittently degraded landmark tracking confidence, producing exactly the kind of boundary-approaching deviation anticipated in this analysis.

**TABLE 3:** Confusion matrix for per-repetition performance classification. Rows: mocap-derived classification (ground truth). Columns: TIAGo-derived classification. Counts pooled across all participants, sessions, and difficulty levels (N=120) .

	TIAGo			
Mocap reference	Fail	Acceptable	Great	Total
Fail	24	0	0	24
Acceptable	0	8	0	8
Great	0	0	88	88
Total	24	8	88	120

### 3.4 Adaptive behavior validation

The adaptive difficulty progression logic operated correctly for all 16 participants, as verified by comparing the system-assigned difficulty level for session 2 against the expected outcome for each group. All adaptive decisions were based on TIAGo-derived session statistics, whose concordance with the BTS reference (Section 3.3) confirms the validity of the decisions.

In the high-performance group (A), all eight participants achieved TIAGo-derived mean session scores well above the promotion threshold in session 1 (group mean $\bar{S}=93.5\pm 6.3$ , range 86.0–100.0, fail ratio = 0.00), triggering automatic promotion to the Hard level (±20°) for session 2. The lower mean relative to the BTS-derived scores is likely attributable to transient occlusion artifacts, but all individual session means remained well above the $\bar{S}=75$ threshold, ensuring correct promotion for every participant. Mean scores in session 2 remained high ( $\bar{S}=95.5\pm 5.7$ , range 86.2–100.0, fail ratio = 0.00), confirming that the onboard scoring system reliably captured high-quality performance also at the increased arc eccentricity of the Hard level.

In the low-performance group (B), all eight participants produced a fail ratio of exactly $r_{\text{fail}}=0.50$ in session 1 (24 of 48 repetitions scoring below 25), meeting the demotion threshold and triggering automatic demotion to the Easy level (±10°) for session 2. The mean session score per participant ( $\bar{S}=41.2\pm 0.8$ , range 39.7–41.8) is consistent with the controlled error pattern (three zero-score repetitions and one S≈50 repetition per participant). Following demotion, all eight participants achieved near-perfect scores in session 2 ( $\bar{S}=99.6\pm 1.0$ , range 97.3–100.0, fail ratio = 0.00), consistent with the lower RMSE observed at the Easy difficulty level (Section 3.1), which reduces the likelihood of artifact-induced score deviations.

The session-level score trajectories for both groups are shown in Figure 4b. It should be noted that the error pattern in session one of the low-performance group was deliberately piloted by the experimenter–all participants were fully capable of performing the task correctly at all difficulty levels, as demonstrated by the correct executions within the same session. The controlled condition was designed to simulate the performance profile expected from a USN patient struggling with the current difficulty level, and validates the demotion logic under a worst-case scenario. The between-session score changes therefore serve primarily as a functional end-to-end verification of the data pipeline, from per-repetition metric computation in the BT through session-level aggregation in SessionAggregator to the difficulty decision in the Coach node.

## 4 Discussion

This study evaluated three prerequisites for the clinical deployment of HemiBot as an autonomous rehabilitation coaching system: the reliability of the onboard kinematic sensing pipeline, the accuracy of the per-repetition scoring metric, and the correctness of the adaptive difficulty progression logic. The results provide positive evidence on all three fronts, while also identifying specific technical limitations that must be addressed before the system can be used with patient populations.

### 4.1 Kinematic sensing

RMSE increased significantly with arc eccentricity for both metrics, with post-hoc differences significant between Easy and Medium and between Easy and Hard, but not between Medium and Hard, suggesting that the dominant contribution to absolute error arises from the transition from the frontal to the lateral workspace rather than accumulating linearly with eccentricity. The concurrent increase in dispersion at higher difficulty levels points to a subset of trials affected by transient wrist occlusions during lateral arm extension, a known failure mode of monocular markerless pose estimation at non-frontal configurations (Dill et al., 2023). Despite this, median RMSE values remain modest across all difficulty levels, so the observed artifacts do not compromise classification accuracy–a point confirmed by the perfect scoring concordance (Section 3.3). The proximity of the wrist to the end effector during active reaching provides a further, task-specific source of occlusion in correctly executed repetitions–an effect expected to be more pronounced in Group A, who followed the end effector actively throughout the arc, as discussed in Section 3.3.

### 4.2 Scoring concordance

It should first be recalled that the objective of this analysis was to verify the fidelity of the onboard, markerless measurement pipeline against the mocap gold standard, specifically, whether the TIAGo-derived score for a given repetition consistently falls in the same performance band as the mocap-derived score, under conditions in which healthy participants were explicitly instructed and piloted to reproduce a targeted performance level (Fail, Acceptable, or Great). The absence of band misclassification across all 120 repetitions confirms that the markerless onboard tracker, despite the transient artifacts reported in Section 3.1, does not introduce errors large enough to displace a repetition from its reference band. In particular, no cross-band error between Fail and Great occurred–the only class of error capable of triggering an incorrect adaptive decision. However, this result should be interpreted in light of the experimental design. The performance conditions tested in this study were centered around distinct clusters ( S≈0 , S≈50 and S≈100 ), maintaining margins of at least 25 points from the classification boundaries at S=25 and S=75 . As visible in Figure 4a, only one repetition approached a decision boundary. Consequently, while these findings establish high classification accuracy for unambiguous motor performance, they do not fully characterize the system’s behavior when performance occurs near the decision thresholds (e.g., S∈[20,30] or S∈[70,80] ). Near these boundaries, even minor measurement perturbations—such as brief wrist occlusions during lateral reaching—could potentially cause boundary jitter. For instance, a genuinely high-quality trial with a true motor performance of S=76 might be misclassified as Acceptable if a transient occlusion drops the TIAGo-derived score to S=74 . While such intra-band shifts (between Great and Acceptable) do not trigger inappropriate demotions, a similar shift near the lower boundary (S=25) could affect the session fail ratio $r_{\text{fail}}$ and prematurely trigger a difficulty reduction. This is consistent with the underlying tracking implementation (Section 2.5): occlusions are not explicitly detected, and a landmark is discarded for a given frame only when it yields an invalid depth reading, in which case the sample is omitted rather than extrapolated by the Kalman filter. Occlusions that still return a valid depth value are instead retained as degraded samples. No minimum-valid-sample threshold is enforced before a repetition is scored. Therefore, an occlusion episode can either reduce the number of samples contributing to a repetition’s score, or inject artifact-affected samples into it, without triggering any explicit invalidation. These are the most plausible mechanisms underlying the boundary-approaching deviation discussed above. To mitigate this risk in clinical deployment, two structural safeguards are built into the architecture. First, the real-time graphical scoreboard (Figure 2) requires therapist validation after each repetition, allowing the clinician to manually override artifact-affected scores prior to BT advancement or Coach node aggregation. Second, progression decisions are driven by session-wide metrics ( $\bar{S}$ and $r_{\text{fail}}$ ) rather than single-repetition triggers, inherently smoothing out isolated tracking anomalies. Furthermore, future iterations of the Coach node will incorporate a decision hysteresis band (e.g., S=75±5 ) or exponential moving averages across consecutive sessions, preventing rapid oscillation between difficulty levels for patients exhibiting borderline performance. (Section 4, Limitations).

### 4.3 Adaptive behavior

The correct adaptive decisions for all 16 participants confirm that the promotion and demotion rules operate as intended. This experiment mainly verifies that the adaptation logic works as intended; its behavior under the more variable performance expected from patients will need to be assessed in future studies. Beyond the algorithmic correctness, this result also validates the reliability of the entire data pipeline: the difficulty decision depends on session-level statistics that are computed by aggregating per-repetition metrics collected across multiple asynchronous ROS 2 nodes, and any failure in this chain would have produced an incorrect adaptive decision. Correct end-to-end behavior across all 16 participants is therefore a non-trivial validation result for a distributed, asynchronous ROS 2 pipeline.

### 4.4 Comparison with related work

Contact-based systems such as the REAplan (Doumas et al., 2025) and the Armule (Chen et al., 2021) implement adaptive difficulty for robot-assisted rehabilitation through physical guidance or resistance. The present system achieves adaptive coaching in a non-contact paradigm, which is particularly relevant for USN patients who may resist physical interaction and for whom exogenous visual stimulation in contralesional space is the primary therapeutic modality. Unlike force-based approaches, which predominantly target the *Aiming* component of the neglect deficit through motor assistance, the present paradigm couples the *Where* (perceptual-attentional) and *Aiming* (motor-intentional) components within a single task, in line with the *Where/Aiming* model (Barrett et al., 2022) and the evidence base for spatiomotor cueing and Limb Activation Treatment (Robertson and North, 1992; Kalra et al., 1997; Robertson et al., 2002). As noted in Somma et al. (2026), this joint attentional-motor approach remains comparatively underexplored in robotic platforms for USN rehabilitation.

Data-driven personalization of exercise difficulty has been validated in exoskeleton-based systems: a retrospective analysis of Armeo Spring session logs across 71 patients showed that task difficulty is a consistent determinant of performance and that disease-specific improvement trajectories can be identified, supporting the use of quantitative performance data to guide progressive difficulty adaptation (Lui et al., 2026). The BT-based architecture offers a complementary modularity advantage: each behavioral node can be independently tested, replaced, or extended–for instance, by substituting HandPointingMetrics with a gaze-aware scoring node once reliable eye-tracking becomes available–without modifying the overall session orchestration logic. A recent systematic review and meta-analysis demonstrated that ML-adaptive virtual reality systems–predominantly using reinforcement learning and deep learning for real-time difficulty adjustment–improve upper limb motor function in stroke survivors by a clinically significant margin (FMA-UE mean difference: +7.47 points, 95%CI: 5.38–9.57, p<0.001 ) (Al Tawil et al., 2026), suggesting that analogous real-time adaptation mechanisms applied to HemiBot’s trajectory parameters represent a natural next step.

The non-contact coaching role of humanoid robots in rehabilitation has been validated from two complementary perspectives. A reliability study using the THER-I-ACT instrument demonstrated that coaching, feedback, and relational therapeutic interactions delivered by a humanoid robot can be comprehensively and reliably assessed (ICC ≥0.90 ) (Platz et al., 2023). A scoping review on Social Assistive Robots in clinical physiotherapy identified coaching, demonstration, monitoring, and peer support as the four primary functional roles (He et al., 2025) – all of which HemiBot addresses through its TTS feedback, moving end-effector, HandPointingMetrics node, and scoreboard respectively. Both reviews highlight the capacity of non-contact social robots to extend therapist reach without physical contact, an argument directly applicable to the deployment scenario envisioned at Fondazione IRCCS Mondino. More advanced adaptive policies–such as reinforcement learning approaches that adjust exercise parameters to individual performance in real time–have been explored for social robot coaching in physiotherapy Bettosi et al. (2025), and represent a natural next step beyond the present threshold-based progression.

A further consideration concerns the target clinical population: transient wrist-occlusion artifacts, identified here as a source of increasing error at larger eccentricities, may affect stroke patients with hemiparesis differently than healthy participants, since atypical or reduced-amplitude limb kinematics could increase the frequency or duration of low-confidence landmark tracking. This potential interaction between motor impairment and markerless tracking reliability was not assessed in the present healthy-volunteer sample and should be characterized in future clinical validation. Relatedly, while the empathetic TTS feedback content was implemented and delivered throughout the sessions, its perceived empathy, appropriateness, and effect on participant engagement were not themselves evaluated in this study; this component should therefore be regarded as an unvalidated element of the clinical-readiness pathway, to be assessed in future work.

### 4.5 Limitations

Several limitations of the current work must be acknowledged. First, the study involved exclusively healthy participants; while the recruited age profile ( 54.5±6.8 years) approximates that of the intended clinical population, the scoring thresholds and band boundaries remain to be validated on post-stroke USN patients, who exhibit substantially different movement patterns, fatigue, and attentional fluctuations. Second, the low-performance group’s error pattern was deliberately piloted by the experimenter, so the demotion validation does not reflect organic patient variability. Because the low-performance group followed a controlled, scripted error pattern (S≈0,50,100) , the scoring logic has not yet been evaluated against naturally occurring, continuous performance gradients near the S=25 and S=75 boundaries. Third, only two sessions per participant were recorded, precluding assessment of longitudinal learning effects. Fourth, the onboard scoring metric remains primarily a motor-kinematic measure: while arm-alignment angle captures *Aiming*-type accuracy and hand–end-effector distance provides an indirect proxy for active engagement, neither constitutes a direct measure of the *Where* (perceptual-attentional) component of the neglect response. Wearable eye-tracking glasses are currently being explored by our group as a robust technique for capturing gaze-based attentional state, yet the development needed for such integration is not mature enough for its use into the present system.

Fifth, while the collision-avoidance and emergency-stop mechanisms are implemented and operate consistently at both the motion-control and session-orchestration levels, they have not yet undergone quantitative safety validation: the software detection latency is bounded by the 30 Hz control loop ( ≈33 ms), but the hardware-side actuator latency and the physical stopping time and distance were not measured. The safety architecture should therefore be regarded as a design provision to be experimentally validated before clinical use, not as a demonstrated safety guarantee.

### 4.6 Future Directions

Planned future work includes: (i) a usability study with healthy participants and patient caregivers, assessed *via* the System Usability Scale (SUS), the User Experience Questionnaire (UEQ), and the Patient Global Impression of Change (PGIC); (ii) a clinical efficacy trial with post-stroke USN patients at Fondazione IRCCS Mondino, incorporating USN-specific outcome measures (Behavioral Inattention Test, Catherine Bergego Scale) and a broader neuropsychological battery; (iii) integration of an eye-tracking system to provide a direct, continuous measure of the *Where* component alongside the motor-kinematic scoring already implemented; (iv) gamification of the exercises to sustain engagement across multi-week training programs; and (v) replacement of the current rule-based adaptive logic with a planning- or machine-learning-based closed-loop controller, in line with the deliberative-layer paradigm for autonomous, long-term session management and difficulty adaptation surveyed in (Tamantini et al., 2025). (vi) Two engineering mitigations could reduce the frequency and impact of wrist occlusions in future iterations of the system. First, replacing the current per-frame Kalman filter, which omits a sample outright whenever the depth reading is invalid, rather than propagating a predicted estimate, with a filter that extrapolates joint position for a bounded number of consecutive missing frames would preserve continuity of the tracked trajectory through brief occlusions. Second, lowering the height of the TIAGo end-effector’s ready/rest configuration and the arc trajectory itself could reduce the geometric overlap between the end-effector and the wrist landmark from the camera’s viewpoint during active reaching, directly reducing the incidence of the occlusion mechanism identified in Section 3.1.

## 5 Conclusion

This paper presented *HemiBot*, a complete ROS 2 software architecture for autonomous, adaptive rehabilitation session management on the TIAGo mobile manipulator, designed to target egocentric peripersonal neglect through a non-contact, robot-driven attentional cueing paradigm. The system integrates a multi-node Behavior Tree for real-time exercise execution and kinematic scoring, a Coach node for session-level adaptive difficulty progression, Italian empathetic TTS feedback, and a therapist-facing scoreboard. A controlled experiment with 16 healthy participants demonstrated three key results. First, kinematic RMSE remained modest across all difficulty levels, despite a statistically significant increase with arc eccentricity attributable to transient wrist occlusions at non-frontal arm configurations. Second, scoring concordance between TIAGo-derived and mocap-derived classifications was perfect (100%, κ=1.000 ) across all 120 repetitions, confirming that the conservative scoring thresholds absorb both natural motor variability and artifact-induced deviations without compromising classification reliability. Third, the adaptive difficulty logic operated correctly for all 16 participants, validating the end-to-end reliability of the data pipeline from per-repetition metric computation through session-level aggregation to the Coach node decision. Together, these results characterize the measurement and decision chain of HemiBot under controlled conditions, and identify the specific analyses–near-boundary scoring behavior and validation on naturally graded performance–required before a clinical trial with post-stroke USN patients can be undertaken.

## Data Availability

The raw data supporting the conclusions of this article will be made available by the authors, without undue reservation.
