# Supplementary material for: HemiBot: design and technical validation of an adaptive Robotic coaching system for Unilateral Spatial Neglect rehabilitation
Source: Front Robot AI. 2026 Oct 21;13:1950821. doi: 10.3389/frobt.2026.1950821 (PMC13730947; doi:10.3389/frobt.2026.1950821)
Supplement: Supplementary file 1 [file DataSheet1.pdf]

Supplementary Material

SUPPLEMENTARY TABLE S1 — ITALIAN TTS FEEDBACK MESSAGES

Table S1 lists all Italian natural-language messages delivered by HemiBot during and at the end of each session. Per-repetition messages are selected probabilistically and uniformly from the pool corresponding to the performance band assigned to the completed repetition (Fail, Acceptable, or Great). Session-level messages are selected once at the end of each session and correspond to the adaptive decision taken by the Coach node (Demotion, Maintain, or Promotion). Messages were authored in consultation with the rehabilitation specialists at Fondazione IRCCS Mondino to ensure clinically appropriate, empathetic, and non-repetitive phrasing. Probabilistic selection among messages within each pool prevents the participant from receiving identical feedback across consecutive repetitions or sessions, reducing habituation effects.

Table S1. Italian TTS feedback messages delivered by HemiBot, stratified by trigger and performance outcome. Per-repetition messages are triggered at the end of each repetition; session-level messages are triggered once at the end of the session. One message is selected uniformly at random from each pool.

Trigger	Message (Italian)
Per-repetition — Fail band ($S < 25$)	
	"Non ti preoccupare, queste cose richiedono tempo. Riproviamo insieme, con calma."
	"Va benissimo, è normale dover ripetere. Ripartiamo, tu ce la fai."
	"Non mollare! Proviamo ancora una volta, cerca di seguire il movimento del braccio."
	"È un esercizio impegnativo. Respira e riproviamo insieme."
	"Nessun problema, ci vuole pratica. Ricominciamo, stavolta andrà meglio."
Per-repetition — Acceptable band ($25 \leq S < 75$)	
	"Stai andando bene, continua così!"
	"Bravo, il movimento è sulla strada giusta. Mantieni questa concentrazione."
	"Bene! Stai prendendo il ritmo, vai avanti così."
	"Il lavoro si vede. Stai migliorando, continua."
	"Ben fatto! La coordinazione sta arrivando, non fermarti."
Per-repetition — Great band ($S \geq 75$)	
	"Ottimo lavoro, davvero! Stai eseguendo il movimento benissimo."
	"Fantastico! Si vede che ti sei impegnato, complimenti."
	"Bravissimo! Il movimento è preciso e fluido, sono molto soddisfatto."
	"Eccellente! Stai dando il massimo e si sente."
	"Meraviglioso! Continua così, stai facendo progressi importanti."
Session-level — Demotion (next session: easier level)	
	"Hai lavorato bene oggi, anche se l'esercizio è stato impegnativo. Per la prossima sessione proveremo con un movimento un po' più contenuto, così puoi consolidare la tecnica con più tranquillità."
Session-level — Maintain (next session: same level)	
	"Ottima sessione! Stai lavorando al ritmo giusto per te. Continuiamo con questa difficoltà anche la prossima volta, per consolidare quello che hai imparato oggi."
Session-level — Promotion (next session: harder level)	
	"Sei stato davvero bravo oggi! Hai gestito l'esercizio con grande sicurezza. La prossima sessione potremmo provare a sfidare un po' di più, ampliando il movimento per continuare a migliorare."
